# Effects of selected feed additives on rumen pH dynamics, physiological responses, and rumen and cecum morphometrics in feedlot beef cattle

**DOI:** 10.1371/journal.pone.0356262

**Published:** 2026-08-19

**Authors:** Maria Betânia Niehues, Daniel Ioan Campos Gomes de Gouvêa, Alexandre Perdigão, Victor Valério Carvalho, Tiago Sabella Acedo, Luis Fernando Monteiro Tamassia, Cyntia Ludovico Martins, Mário De Beni Arrigoni

**Affiliations:** 1 School of Veterinary Medicine and Animal Science, Sao Paulo State University, Botucatu, São Paulo, Brazil; 2 DSM-Firmenich, Department of Innovation and applied Science, São Paulo, Brazil; 3 DSM-Firmenich, Animal Nutrition and Health, Basel, Switzerland; Tokat Gaziosmanpaşa University: Tokat Gaziosmanpasa Universitesi, TÜRKIYE

## Abstract

The search for alternatives to ionophore antibiotics such as monensin has increased due to regulatory restrictions and consumer demand for natural feed additives. The objective of this study was to evaluate the effects of feed additive combinations compared with sodium monensin on performance, carcass traits, ruminal and cecal health, and meat quality in feedlot-finished cattle. Twenty-four F1 Angus–Nellore crossbred bulls (BW = 456 ± 21.77 kg) were randomly assigned to one of three treatments: 1) sodium monensin (**MON**; 26 mg/kg DM); 2) a blend of essential oils plus α-amylase (**BEO**, 90 and 560 mg/kg of DM, respectively); and 3) BEO + 25-hydroxicholecalciferol (**BEO + HyD**; 1 mg/animal/day). Animals were considered the experimental units, and data were analyzed using a mixed model with treatment as fixed effect and animal as random effect. Feeding BEO and BEO + HyD increased DMI (P < 0.0001), HCW (P = 0.01), and dressing percentage (P = 0.03), and tended to increase ADG (P = 0.07) and final BW (P = 0.08) compared with MON, with no differences in feed efficiency (P > 0.50). Feed additives did not affect most meat quality traits (P > 0.05). During the adaptation period and overall, animals fed BEO and BEO + HyD maintained higher mean and minimum ruminal pH, spent less time below pH 6.2 and 6.0, and had a lower area under the curve below these thresholds, along with lower ruminal temperature compared with MON (P ≤ 0.05). Additionally, over the entire feeding period, animals fed BEO and BEO + HyD spent less time below pH 5.8 (P = 0.02). Animals fed MON and BEO exhibited greater ruminal absorptive surface area (P = 0.007) than those fed BEO + HyD. Cecal morphometric parameters were not affected by treatments (P > 0.05). In conclusion, the combination of a blend of essential oils and α-amylase improved ruminal health and enhanced performance and carcass production without affecting meat quality or cecal morphology compared with monensin. Supplementation with 1 mg of 25-(OH)D_3_ showed limited additional effects on carcass traits.

## Introduction

The inclusion level of concentrate in the finishing diets in Brazil has increased, on a dry matter (**DM**) basis, from 71.2% [1] to 83.3% [2]. This rise in the use of concentrate feedstuffs in feedlot diets is associated with higher inclusion of cereal grains and investments in more extensive corn processing methods to enhance starch availability and the energy content of the final diets [3]. However, this dietary strategy to improve feedlot performance is often linked to the development of metabolic disorders, such as ruminal acidosis, which is the most prevalent digestive disorder in feedlot cattle in North America [4] and Brazil [2].

In this context, the use of monensin (MON) in livestock production systems is supported by several years of research demonstrating its benefits, primarily through the modulation of rumen fermentation [5,6], contributing to a lower incidence of metabolic disorders [7] while improving feed efficiency [8]. However, due to concerns about the development of bacterial resistance, the use of these antibiotic ionophores in animal feed is limited or banned in many countries [9]. Recently, this has generated significant efforts to develop alternatives that can enhance the rate, efficiency, and quality of gain and performance while preventing certain metabolic disorders and ensuring food safety security.

Alternative compounds, such blend essential oils (EO) have been evaluated as alternative feed additives in finishing cattle diets due to their potential to enhance ruminal fermentation efficiency and animal productivity [10,11]. Among the most studied compounds are limonene, thymol, vanillin, eugenol, capsaicin, and cinnamaldehyde [12]. These plant-derived secondary metabolites are mainly extracted by steam distillation [13]. Because of their bioactive properties, particularly antimicrobial effects, EO represent a promising alternative to antibiotics for modulating the rumen microbiome through selective inhibition of microbial populations [14]. According to McIntosh et al. [15], combining different EO can decrease both the abundance and diversity of hyper-ammonia-producing bacteria, thereby reducing deamination activity and ammonia production in the ruminal environment.

Furthermore, supplementing feedlot diets with exogenous α-amylase (**AM**) has been investigated as a strategy to enhance starch utilization [16], although responses in animal performance have been variable [17,18]. In dairy systems, AM supplementation has been associated with improvements in starch degradation, milk yield, and energy balance [19,20]. In beef cattle, the use of AM in combination with essential oils (**BEO**) has been explored as a means of modulating rumen fermentation patterns, potentially improving growth performance and meat quality [21,22]. The association of BEO resulted in animals with heavier carcasses compared to those fed MON, due to higher energy intake and greater nitrogen uptake [23].

Additionally, 25-hydroxicholecalciferol (25(OH)D_3_) has been proposed to enhance dressing percentage [24] and carcass [25] due to increased expression of genes associated with muscle anabolism [24]. Previous studies have observed that cattle supplemented with EO + 25(OH)D_3_ (**HyD**) had greater hot carcass weight (**HCW**) and *longissimus muscle* (**LM**) area than those fed MON + Virginiamycin (**VM**; [26]. Mendoza-Cortéz et al. [27] reported greater dry matter intake (**DMI**) and performance in cattle fed the EO + HyD combination compared to those fed MON. Conversely, [28] did not observe differences in performance and carcass production between cattle fed EO + HyD and those fed MON.

Since there are no studies in the literature evaluating the effect of EO combined with AM and HyD in feedlot diets containing high levels of starch, it was hypothesized that the combination of EO, AM, and HyD would improve the rumen environment and positively impact animal performance and carcass production. Therefore, the objective of this study was to evaluate the combination of EO and AM, as well as the association of EO with AM and HyD in feedlot diets with high starch content, on the performance, carcass traits, ruminal and cecal health, and meat quality of feedlot F1 Angus-Nellore crossbred cattle.

## Materials and methods

The study was conducted at the feedlot of the Innovation Center Tortuga, DSM Nutritional Products (Rio Brilhante, Mato Grosso do Sul, Brazil). Animal care and handling procedures were performed in accordance with the guidelines of the Animal Use Ethics Committee (CEUA) and were approved by the Ethics Committee on Animal Use of the Innovation Center Tortuga, DSM Nutritional Products (Protocol No. CEUA 003/2019).

### Animals and Treatments

Twenty-four F1 Angus-Nellore yearling bulls (18 months old, 456 ± 21.77 kg), originating from a grazing system, were randomly assigned to a completely randomized design with three treatments and eight replicates, with the individual animal considered the experimental unit. The animals were housed in a collective pen with a total area of 810 m² (33.75 m²/animal) and assigned to the following experimental treatments: T1) sodium monensin (MON; 26 mg/kg DM; Rumensin®, Elanco Animal Health, Indianapolis, IN, USA); T2) a blend of essential oils plus α-amylase (BEO; 90 and 560 mg/kg DM, respectively; CRINA Ruminants®, DSM Nutritional Products, Basel, Switzerland), containing thymol, eugenol, limonene, and vanillin, and an exogenous α-amylase enzyme (RONOZYME Rumistar®, DSM Nutritional Products); and T3) BEO supplemented with 25-hydroxycholecalciferol (BEO + HyD; 1 mg/animal/day; Hy-D®, Rovimix 1.25%, DSM Nutritional Products). The inclusion levels of BEO and AM were based on previous feedlot studies demonstrating their efficacy [21,29], whereas the dosage of 25-(OH)D_3_ was based on Martins et al., [24].

Individual DMI was measured daily using the Intergado monitoring system (Ponta Agro, Betim, Minas Gerais, Brazil), which consists of individual electronic feed bunks equipped with head gates. At the beginning of the experiment, animals were fitted with an ear tag containing a unique passive transponder (FDX—ISO 11784/11785; Allflex, Joinville, SC, Brazil), allowing access only to their designated automatic feed bunks.

Individual animal DMI was calculated daily based on feed bunk records and the dry matter (DM) content of the diet, which was determined daily by collecting samples of the total diet from each treatment and drying them in a MARCONI oven at 105 °C for 24 h. Diets were offered ad libitum, aiming to maintain feed refusals between 3% and 5% of the amount offered on the previous day, ensuring adequate intake while minimizing feed waste.

### Feeding and Management Description

At the beginning of the study, all animals were individually weighed and subjected to a health management protocol, including vaccination against clostridiosis (Bovilis® Poli-Star®, Merck & Co., Inc., Rahway, NJ, USA) and deworming with Albendathor (JA Saúde Animal, Brazil).

Because the health and management history of the animals was unknown, they underwent a 15-day adaptation phase aimed at stabilizing ruminal microbiota and allowing acclimatization to the experimental facilities, automatic feed bunks, and routine management practices. Following this period, cattle underwent a 14-day dietary adaptation phase, during which they received step-up diets (adaptation diets 1, 2, and 3) for 5, 4, and 5 days, respectively. From day 15 of the trial until slaughter, animals were fed a finishing diet containing 90% concentrate. This adaptation strategy was based on the methodology described by Parra et al. [30], with adjustments for the present study. Cattle were fed for 102 days, and diets were offered ad libitum twice daily at 10:00 a.m. and 3:00 p.m., with free access to a water trough (650 L capacity; 3.00 × 0.80 × 0.20 m).

Diets formulation were based on the Large Ruminant Nutrition System (LRNS, Tedeschi and Fox, [31]), which estimates nutrient requirements and performance responses of beef cattle (Table 1). The experimental diets consisted of sugarcane bagasse, ground corn, soybean hulls, cottonseed, soybean meal, a mineral-vitamin supplement, urea and additives.

**Table 1:** Ingredient and chemical composition of diet (DM basis).

Item	Diets			
	STEP1	STEP2	STEP3	Finisher
**Ingredients,%**				
**Sugarcane bagasse**	25.00	20.00	15.00	10.00
**Soybean meal**	7.00	7.50	7.50	7.50
**Ground corn** ^**a**^	41.00	56.50	67.00	77.50
**Soybean hulls**	16.00	6.00	2.50	–
**Cottonseed**	6.00	5.00	3.00	–
**Mineral and vitamin supplement** ^**b, c**^	5.00	5.00	5.00	5.00
**Nutrient content, %DM**				
**Dry Matter**	53.50	59.00	61.00	68.00
**Total digestible nutrients** ^**d**^	69.00	74.00	77.00	81.00
**Crude Protein**	14.60	14.50	14.40	14.20
**Neutral detergent fiber**	39.31	33.50	26.67	18.30
**Starch** ^**e**^	29.11	40.12	47.57	55.03
**Physically effective NDF** ^**f**^	31.00	23.00	19.00	15.00
**NEg, Mcal/kg** ^**g**^	1.01	1.13	1.22	1.31

^a^Medium ground (1.70 mm). ^b^Mineral and vitamin supplement containing dietary treatments: MON = sodium monensin (26 mg/kg DM); BEO = blend of essential oils + exogenous α-amylase (90 and 560 mg/kg DM, respectively); BOE + HyD = blend of essential oils + exogenous α-amylase + 25- hydroxycholecalciferol (90, 560 mg/kg mg/kg DM and 1 mg/animal/day, respectively). Sodium monensin (Rumensin) was from Elanco Animal Health, Indianapolis, IN. The blend of essential oils (CRINA Ruminants), and the exogenous enzymes (α-amylase [RONOZYME RumiStar] and 25-hydroxycholecalciferol (Rovimix Hy-D 1.25%) were provided by DSM Nutritional Products, Basel, Switzerland. ^c^Mineral and vitamin supplement was composed (DM basis) of 700 g NPN/kg, 130 g/kg Ca, 12 g/kg P, 27 g/kg S, 15 g/kg Mg, 25 g/kg K, 42 g/kg Na, 6 mg/kg Co, 400 mg/kg Cu, 5,00 mg/kg Cr, 20 mg/kg I, 800 mg/kg Mn, 5.00 mg/kg Se, 1.500 mg/kg Zn, 125,000 UI/ kg vitamin A, 12,500 UI/kg vitamin D_3_, 1,300UI/kg vitamin E, 67 mg/kg biotin, 2.0 × 10^9^ UFC/kg *Saccharomyces cerevisiae*. ^d,e,g^Estimated by equations proposed by Large Ruminant Nutrition System [31]. ^f^Physically effective NDF determined according to the method described by Heinrichs and Kononoff [32].

For bromatological analyses, samples of feed ingredients and experimental diets from each treatment were collected weekly, properly identified, and stored at −20 °C. At the end of the experimental period, samples were thawed, composited by treatment and experimental period. Subsequently, samples were analyzed for dry matter (DM) and crude protein (CP) according to AOAC [33] method 976.05), and neutral detergent fiber corrected for ash (NDF), following Mertens [34], using heat-stable α-amylase and without sodium sulfite.

### Data collection of ruminal pH

The devices used to measure individual ruminal pH and temperature were smaXtec bolus (smaXtec Animal Care GmbH, Graz, Austria). All procedures for boluses calibration, administration, and data monitoring followed the protocol described by Crossland et al. [35]. Briefly, on day 0, eight animals from each treatment were randomly selected to receive an oral bolus. Prior to administration, all boluses were calibrated according to the manufacturer’s instructions using a pH 7 buffer solution for at least 5 minutes and then administered using a dosing gun. Two additional boluses were provided by the manufacturer in case of calibration failure.

Data from all smaXtec boluses were continuously recorded telemetrically throughout the experimental period, generating daily pH and temperature measurements at 10-minute intervals. At the end of the experiment, all data were retrieved using smaXtec Messenger® software and processed in Microsoft Excel. Individual parameters were calculated for each animal, including minimum, mean, and maximum pH, area under the curve, duration of pH below 6.2, 6.0, and 5.8, and mean ruminal temperature.

### Serum concentrations of calcium, phosphorus and 25-(OH)D_3_

Blood samples were collected from the caudal vein of four animals per treatment at the beginning and end of the experimental period. For blood collection, 25 × 0.8 mm vacutainer needles (Vacuplast, Shandong, China) and vacuum tubes containing a clot activator (BD Vacutainer®, Franklin Lakes, NJ, USA) were used. After collection, samples were immediately placed on ice until centrifugation (approximately 30 min). Samples were centrifuged at 3,000 × g for 15 min, and a 0.5 mL aliquot of serum was transferred to Eppendorf tubes. Serum samples were then stored at −20 °C until analysis.

Serum concentrations of 25-(OH)D₃ and ionized calcium (iCa) were determined by chemiluminescence and dry chemistry methods, respectively (Oswaldo Cruz Clinical Analysis Laboratory, Taubaté, SP, Brazil). Total calcium (tCa) and total phosphorus (tP) concentrations were determined using commercial kits (Bioclin Biocontrol) based on endpoint colorimetric methods—Arsenazo III for calcium and UV phosphomolybdate for phosphorus (Veterinary Clinical Laboratory, FMVZ, UNESP, Botucatu, SP, Brazil).

### Apparent Total Tract Starch Digestibility

Fecal samples were collected from all animals (n = 24) once daily at 13:00 h for five consecutive days (days 70–74 of the finishing period). The collection procedure followed the recommendations of Rigueiro et al. [36], whereby feces were collected directly from the ground immediately after defecation using labeled plastic bags. Additionally, samples of the offered diet and feed refusals were collected and stored together with the fecal samples at −20 °C for subsequent laboratory analyses.

Starch concentration in the diet, feed refusals, and fecal samples was determined according to the methods described by Pereira and Rossi [37] and Hendrix [38], whereas crude protein was determined using the Kjeldahl method [39]. Starch digestibility was then calculated as described by Zinn et al. [40].

### Feedlot performance and carcass traits

Body weight (BW) and average daily gain (ADG) were monitored daily using a Bosch® Precision Livestock platform installed in the feedlot pen. At the beginning of the experiment, all animals were fitted with a Bosch electronic ear tag, allowing individual identification. For performance evaluation, individual BW measurements recorded by the Bosch® Precision Livestock platform at the beginning (initial BW), day 28 (d28 BW), and the end of the experimental period (final BW) were used. All BW values were adjusted by subtracting 4% to account for shrunk body weight.

ADG was calculated as the difference between final and initial BW divided by the number of days in each experimental period. DMI expressed as a percentage of BW (DMI, %BW) was calculated by dividing daily DMI by BW and multiplying the result by 100. Feed efficiency (G:F) was calculated by dividing ADG by daily DMI. The 12th-rib fat thickness, Biceps femoris fat thickness, LM area, and marbling were measured by ultrasound at the end of the experimental period following the method described by Perkins et al. [41]. Images were collected using an Aloka SSD-1100 Flexus RTU unit (Aloka Co. Ltd., Tokyo, Japan) equipped with a 17.2 cm, 3.5 MHz probe.

At the end of 102-day feedlot period, animals were transported 263 km to a commercial abattoir (Bataguassu, MS, Brazil). Animals were slaughtered under official inspection in accordance with Brazilian regulations for animal welfare and humane slaughter (Ordinance No. 365/2021, Ministry of Agriculture, Livestock and Food Supply). Prior to exsanguination, animals were rendered unconscious by mechanical stunning using a captive bolt device, ensuring rapid loss of sensibility and absence of pain. All procedures were performed by trained personnel, and efforts were made to minimize stress, discomfort, and suffering during handling and slaughter.

After removing the kidney, pelvic, and heart fat, HCW was recorded. Dressing percentage was calculated as the ratio of HCW to final BW.

### Muscle fiber area

To determine the average muscle fiber area, samples of the *Longissimus thoracis* (LT) muscle from the left half carcasses of all animals were collected shortly after slaughter. After collection, muscle samples were sectioned into fragments approximately 1.0 cm in length and 0.5 cm in thickness, embedded in OCT compound, placed in cryogenic tubes, and rapidly frozen in liquid nitrogen.

Tissue quality was assessed during histological processing based on the preservation of muscle fiber morphology, including clear fiber boundaries, polygonal fiber shape, and the absence of evident freezing artifacts such as vacuolization, fiber disruption, or extensive ice crystal formation. Only sections with adequate structural preservation were used for morphometric analyses. Histological procedures were performed following the methodology described by Costa et al. [42], with minor modifications. Briefly, tissue sections were obtained using a cryostat, stained with hematoxylin and eosin (H&E), and examined under a light microscope equipped with an image capture system (Leica Qwin Image Analyzer, McBain Systems, CA, USA). To determine muscle fiber area, 200 fibers per animal were measured [43] by ImageJ software.

### Meat quality analysis

Samples of the LT muscle were collected during carcass deboning (24 h post-mortem) from the left half-carcasses between the 11th and 13th ribs of each animal [44]. The samples were vacuum-packed in polyethylene bags and stored at −20 °C until further analyses. Three steak samples (approximately 2.54 cm thick) were obtained from each animal: one for pH and color determination, another for shear force and cooking loss evaluation, and a third for proximate composition analysis, as described below.

For pH and color determination, samples were removed from vacuum packaging and exposed to oxygen for 30 min at 4 °C. Meat pH was measured using a digital pH meter (Model AK86, AKSO Instruments, Rio Grande do Sul, Brazil) calibrated with standard buffers (pH 4.0 and 7.0). Color parameters (L* = lightness, a* = redness, b* = yellowness) were measured on the same steak according to the CIELab system using a portable colorimeter (CR-400, Konica Minolta, Tokyo, Japan), as described by [45]. Chroma index was calculated as $\sqrt{(a*{)}^{\text{2}}+(b*{)}^{\text{2}}}$ and the hue angle was determined using the formula $tan\left(\frac{b*}{a*}\right)$^-1^ [46, 47 respectively].

Meat shear force (SF) was evaluated according to the protocol described by Wheeler et al. [48] with minor modifications. Steak samples were thawed under refrigeration (4 °C) for approximately 24 h, weighed, and cooked in an industrial electric oven until the internal temperature reached 71 °C. After cooking, samples were cooled at 4 °C for 12 h, and eight cylindrical cores (approximately 12 mm in diameter) were removed parallel to the muscle fiber direction. Shear force was determined using a Brookfield CT-3 Texture Analyzer (AMETEK Brookfield) equipped with a Warner–Bratzler shear blade. Results were expressed in kilograms (kg) as the mean of eight measurements per sample.

Cooking losses were determined using the same procedure described for shear force. Steaks were weighed before and after cooking and cooled at room temperature (25 °C). Cooking loss was calculated as the percentage difference between raw and cooked weights, according to Baldassini et al. [49].

Analysis of centesimal composition was performed according to Santiago et al. [50], with minor adaptations. Steaks were thawed under refrigeration (4 °C) for 24 h, and visible subcutaneous fat and connective tissue were removed from all samples. Approximately 180 g of minced and homogenized sample (processed for 5 min in a food processor) was used to determine moisture, protein, and fat contents by near-infrared spectroscopy (NIRS) using a FoodScan Lab™ instrument (Foss NIRSystems, Inc., USA), following the procedure described by Anderson [51]. After each reading, the sample was removed, rehomogenized, and reanalyzed, and the mean of three readings was recorded [52]. Ash content was determined according to AOAC [53].

### Muscle analyses of calcium and phosphorus

Calcium (Ca) and phosphorus (P) analyses in the LT muscle were performed at the Bioanalytical and Metalloproteomics Laboratory (UNESP, Botucatu, SP, Brazil) by flame atomic absorption spectrometry and visible spectrophotometry, respectively, following acid mineralization of the samples. The mineralization procedure was based on Neves et al. [54] with minor modifications, as briefly described below. Approximately 100 mg of each sample was transferred to 25 mL digestion flasks. Subsequently, 2.00 mL of 14 mol L ⁻ ¹ nitric acid and 0.50 mL of 30% (w/w) hydrogen peroxide were added. After cooling to room temperature, the acid extracts (final volume of approximately 6.50 mL) were transferred to 25.00 mL volumetric flasks, and the volume was brought to completion with ultrapure water. Ca concentrations were determined using a Shimadzu AA-6800 spectrometer equipped with a deuterium background correction system (SR), under the operating conditions described by Neves et al., [54]. P concentrations were determined using the vanadomolybdophosphoric acid spectrophotometric method. This method is based on the reaction of orthophosphate ions in an acidic medium with molybdate and vanadate ions, resulting in the formation of a yellow heteropolyacid complex. This complex absorbs visible radiation at 420 nm, and the absorbance is directly proportional to the concentration of orthophosphate (H_2_ PO_4_^2-^) and/or total phosphorus in the acid extracts [55].

### Muscle and liver gene expression

LT muscle and liver samples for gene expression analysis were collected from 24 animals (8 per treatment) immediately post-slaughter at the abattoir. Samples were placed in labeled cryogenic tubes, snap-frozen in liquid nitrogen, and stored at −80 °C until analysis.

Total RNA was extracted using TRIzol reagent (Invitrogen Co., Carlsbad, CA, USA) following the manufacturer’s instructions. RNA concentration was determined using a NanoDrop OneC spectrophotometer (Thermo Scientific, Carlsbad, CA, USA), and RNA integrity was assessed by electrophoresis on 2% agarose gels based on the visualization of 28S and 18S rRNA bands. The isolated RNA was treated with DNase I (Thermo Scientific, Carlsbad, CA, USA) to remove genomic DNA contamination. RNA quality was considered adequate for downstream analyses, with 260/280 ratios of 2.04 ± 0.01 for muscle samples and 2.08 ± 0.04 for liver samples.

Complementary DNA (cDNA) was synthesized using the High-Capacity cDNA Reverse Transcription Kit (Applied Biosystems™, Cat. No. 4368814, Foster City, CA, USA) according to the manufacturer’s protocol. Primer pairs for eighteen target genes were designed using Primer3Plus software [56] and their specificity was verified using NCBI Primer–BLAST [57] (Tables S1–S3). The target genes included phosphoenolpyruvate carboxykinase 1 (PCK1), phosphoenolpyruvate carboxykinase 2 (PCK2), glucose-6-phosphatase catalytic subunit (G6PC), carbamoyl-phosphate synthetase 1 (CPS1), glutamic-oxaloacetic transaminase 1 (GOT1), argininosuccinate synthetase 1 (ASS1), serum amyloid A3 (SAA3), toll-like receptor 4 (TLR4), TNF receptor-associated factor 6 (TRAF6), mechanistic target of rapamycin (mTOR), ribosomal protein S6 kinase A1 (RPS6KA1), insulin-like growth factor 1 (IGF1), insulin-like growth factor 2 (IGF2), myostatin (MSTN), vitamin D receptor (VDR), cytochrome P450 family 27 subfamily B member 1 (CYP27B1), sterol regulatory element-binding transcription factor 1 (SREBF1), peroxisome proliferator-activated receptor gamma (PPARG), as well as and two reference genes: beta-actin (ACTB) and glyceraldehyde-3-phosphate dehydrogenase (GAPDH).

qPCR reactions were performed on a CFX96™ thermal cycler (Bio-Rad) in a final volume of 10 μL, containing 2 × qPCRBIO SyGreen Mix (PCR Biosystems, London, UK), 1 μL of cDNA, and forward and reverse primers at optimized concentrations. Amplification conditions for ACTB, GAPDH, G6PC, PCK1, SAA3, TLR4, TRAF6, mTOR, MSTN, VDR, IGF2, GOT1, CPS1, CYP27B1, PPARG, and SREBF1 consisted of an initial denaturation at 95 °C for 2 min, followed by 40 cycles of 95 °C for 5 s and 60 °C for 30 s. For PCK2, amplification was performed with an initial denaturation at 95 °C for 2 min, followed by 40 cycles of 95 °C for 5 s, 60 °C for 30 s, and 83 °C for 10 s. For ASS1, IGF1, and RPS6KA1, cycling conditions consisted of an initial denaturation at 95 °C for 2 min, followed by 40 cycles of 95 °C for 5 s, 60 °C for 30 s, and 78 °C for 10 s. Melting curve analysis was performed to confirm single-product amplification. All samples were run in triplicate from the same RNA extraction, and the mean of the triplicates was used for analysis.

qPCR efficiency for each gene was determined individually from the slope of a standard curve generated using triplicate serial dilutions of cDNA. Amplification efficiency (E) was calculated according to the equation: E = 10^(−1/slope)^ [58]. Relative quantification (RQ) of gene expression was calculated using a modified Pfaffl method [58], applying the formula: E _target_^ΔCt target (calibrator Ct – Sample Ct)^. Relative expression values were normalized to the geometric mean of the reference genes ACTB and GAPDH, as previously described for hepatic and muscle tissues [59,60].

### Liver Abscess, Rumen and Cecum Morphometrics

During slaughter, all livers were inspected for the presence of hepatic abscesses. When detected, abscesses were classified according to the scoring system proposed by Brink et al. [61]. To evaluate rumen health, all rumens were emptied, opened, and washed with running water immediately after slaughter to remove digesta. The ruminal epithelium was then examined for lesions, including epithelial disruptions, papillary hyperkeratosis, inflammation, and changes in papillae or epithelial color, following the criteria described by Bigham and McManus [62]. Two trained evaluators, blinded to treatments, independently scored each rumen, and the mean score per animal was used for statistical analysis.

Immediately after rumen evaluation, tissue samples (1 cm^2^) were collected from the dorsal cranial sac and preserved in phosphate-buffered saline (PBS) solution for subsequent macroscopic as described by Resende Júnior et al. [63]. The number of papillae per cm^2^ of ruminal wall (NOP) was manually counted by four independent observers, and the mean value was used for analysis. Subsequently, twelve papillae were randomly selected and sectioned at the base for digital imaging and determination of the average papillary area (APA) using the ImageTool software (UTHSCSA; v2.01). The absorptive surface area (ASA) was estimated using the equation 1+(NOP*MPA)−(NOP*0.002),  according to the methodology described by Daniel et al. [64], and the results were expressed in cm^2^.

For microscopic morphometric analyses, an approximately 1 cm^2^ sample was collected from the ventral cranial sac of each rumen, rinsed in PBS) to remove residual digesta and maintain tissue hydration, and subsequently fixed in 4% paraformaldehyde until histological processing according to standard procedures [65]. Histological measurements, including papillae height, width, surface area, and keratinized layer thickness, were obtained from 10% of the previously determined NOP using a Leica Qwin Image Analyzer coupled to a Leica light microscope.

Cecal evaluation was performed immediately after slaughter, following evisceration, emptying, and washing. The presence of inflammation and lesions in the cecal wall was scored on a scale from 0 (no lesions) to 10 (severe lesions), as adapted from Pereira et al. [66]. Scoring was conducted independently by two trained evaluators blinded to treatments, and the final value represented the mean of both scores.

For histological analyses, a 1 cm^2^ tissue fragment was collected from the central region of each cecum and fixed in 4% paraformaldehyde-buffered solution until processing [67]. Histological procedures were performed as described by Rigueiro et al. [68], including dehydration, paraffin embedding, sectioning, and H&E staining. Morphometric measurements, such as crypt depth and goblet cell count, were obtained from 10% of the total crypts per animal using a Leica Qwin Image Analyzer attached to a Leica light microscope.

### Statistical Analysis

All analyses were performed using the MIXED procedure of SAS (SAS Inst. Inc., Cary, NC), with animals considered the experimental units. The statistical model included treatment as a fixed effect and animal as a random effect. Initial BW and baseline blood values were included as covariates when significant. Prior to the main analysis, data normality was assessed using the PROC UNIVARIATE procedure, and normality was assumed when the Shapiro–Wilk test resulted in P ≥ 0.05. Model adequacy was evaluated by residual analysis to ensure that the assumptions of normality and homoscedasticity were met. When treatment effects were significant (P ≤ 0.05) or tended to be significant (0.05 < P ≤ 0.10), means were compared using the Tukey–Kramer test.

## Results

### Dry matter intake and performance

DMI and productive performance data are presented in Table 2. During the first 28 days of the feeding period, animals fed diets containing BEO and BEO + HyD had greater DMI (P < 0.0001) and DMI as a percentage of BW (P < 0.001); however, no differences were observed for ADG (P = 0.16), BW (P = 0.17), or G:F ratio (P = 0.79).

**Table 2:** Effects of feed additives and their combinations on dry matter intake and productive performance of feedlot F1 Angus-Nellore bulls.

Item^a^	Treatments^b^			SEM^c^	*P*-value^d^
	MON	BEO	BEO + HyD		
**Performance**					
**Initial BW, kg**	453.72	453.14	462.37	6.37	0.30
**Adj. BW d28, kg**	498.89	506.84	512.32	4.50	0.17
**Final BW, kg**	628.04^b^	654.04^a^	651.50^a^	8.44	0.08
**Days 0–28**					
**DMI, kg**	10.47^b^	12.60^a^	12.54^a^	0.25	<.0001
**DMI %BW**	2.19^b^	2.62^a^	2.59^a^	0.05	<.0001
**ADG, kg**	1.53	1.81	1.99	0.16	0.16
**G:F, kg/kg**	0.147	0.145	0.157	0.001	0.79
**Days 0–102**					
**ADG, kg**	1.68^b^	1.94^a^	1.92^a^	0.08	0.07
**DMI, kg**	10.77^b^	13.18^a^	12.82^a^	0.27	<.0001
**DMI %BW**	1.99^b^	2.38^a^	2.31^a^	0.04	0.003
**G:F, kg/kg**	0.156	0.147	0.150	0.005	0.50

a BW = body weight; Adj. BW = adjusted by subtracting 4% to account for shrunk body weight; DMI = dry mater intake; ADG = average daily gain; G:F = calculated as the ratio of ADG to DMI).

b MON = sodium monensin (26 mg/kg DM); BEO = blend of essential oils + exogenous α-amylase (90 and 560 mg/kg DM, respectively); BOE + HyD = blend of essential oils + exogenous α-amylase + 25- hydroxycholecalciferol (90, 560 mg/kg mg/kg DM and 1 mg/animal/day, respectively). Sodium monensin (Rumensin) was from Elanco Animal Health, Indianapolis, IN. The blend of essential oils (CRINA Ruminants), and the exogenous enzymes (α-amylase [RONOZYME RumiStar] and 25- hydroxycholecalciferol (Rovimix Hy-D 1.25%) were provided by DSM Nutritional Products, Basel, Switzerland.

c SEM: standard error of the mean.

d *P* ≤ 0.05 values were considered significant effects and trends were considered at 0.05 *< P ≤* 0.10. a-d: Means with different letters in the same row differ (*P <* 0.05).

During the overall feedlot period (102 days), animals fed BEO and BEO + HyD showed greater DMI (P < 0.0001) and DMI as a percentage of BW (P = 0.003), and tended to have greater ADG (P = 0.07) and final BW (P = 0.08) compared with MON, although no significant differences were observed for G:F ratio (P = 0.50).

### Carcass and meat quality traits

**Fig 1:**
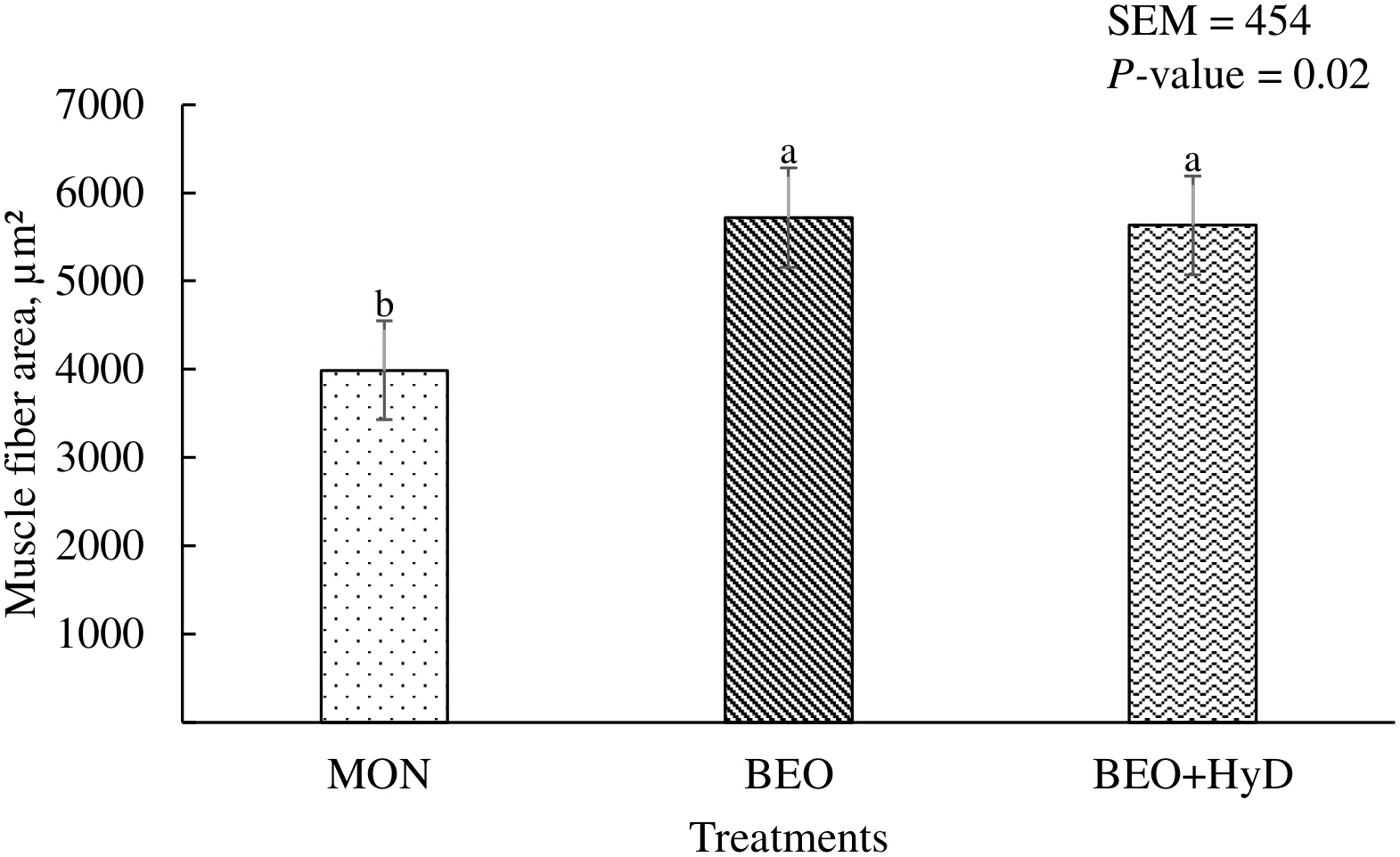
Mean cross-sectional area of *Longissimus thoracis* muscle fibers in feedlot F1 Angus–Nellore bulls. ^a^MON = sodium monensin (26 mg/kg DM); BEO = blend of essential oils + exogenous α-amylase (90 and 560 mg/kg DM, respectively); BEO + HyD = blend of essential oils + exogenous α-amylase + 25- hydroxycholecalciferol (90, 560 mg/kg mg/kg DM and 1 mg/animal/day, respectively). Sodium monensin (Rumensin) was from Elanco Animal Health, Indianapolis, IN. The blend of essential oils (CRINA Ruminants), and the exogenous enzymes (α-amylase [RONOZYME RumiStar] and 25- hydroxycholecalciferol (Rovimix Hy-D 1.25%) were provided by DSM Nutritional Products, Basel, Switzerland. ^b^SEM: standard error of the mean. ^c^*P* ≤ 0.05 values were considered significant effects and trends were considered at 0.05 *< P ≤* 0.10. a-c: Means with different letters in the same row differ (*P <* 0.05).

Animals fed BEO and BEO + HyD showed greater HCW (P = 0.01) and dressing percentage (P = 0.03) compared with those fed MON (Table 3). However, longissimus muscle area, 12th-rib backfat thickness, P8 fat thickness, and marbling were similar among treatments (P > 0.05). Furthermore, feeding BEO and BEO + HyD increased mean muscle fiber area (Fig 1) compared with animals fed MON alone. Representative histological images illustrating muscle fiber morphology for each treatment are provided in the Supporting Information (S4 Fig).

**Table 3:** Effects of feed additives and their combinations on carcass traits of feedlot F1 Angus-Nellore bulls.

Item^a^	Treatments^b^			SEM^c^	*P*-value^d^
	MON	BEO	BEO + HyD		
**Final HCW, kg**	344.13^b^	368.53^a^	371.87^a^	6.41	0.01
**Dressing, %**	55.30^b^	56.66^a^	57.38^a^	0.51	0.03
**LM area, cm** ^**2**^	74.29	77.83	81.14	2.76	0.43
**12th-rib backfat thickness, mm**	6.11	6.83	6.40	0.60	0.69
**P8 fat thickness, mm**	7.87	8.81	8.19	0.84	0.72
**Marbling, %**	2.96	2.65	2.74	0.14	0.31

a HCW = Hot carcass weight; Dressing = calculated as the ratio of HCW to final shrunk BW.

b MON = sodium monensin (26 mg/kg DM); BEO = blend of essential oils + exogenous α-amylase (90 and 560 mg/kg DM, respectively); BOE + HyD = blend of essential oils + exogenous α-amylase + 25- hydroxycholecalciferol (90, 560 mg/kg mg/kg DM and 1 mg/animal/day, respectively). Sodium monensin (Rumensin) was from Elanco Animal Health, Indianapolis, IN. The blend of essential oils (CRINA Ruminants), and the exogenous enzymes (α-amylase [RONOZYME RumiStar] and 25- hydroxycholecalciferol (Rovimix Hy-D 1.25%) were provided by DSM Nutritional Products, Basel, Switzerland.

c SEM: standard error of the mean.

d *P* ≤ 0.05 values were considered significant effects and trends were considered at 0.05 *< P ≤* 0.10. a-c: Means with different letters in the same row differ (*P <* 0.05).

No significant differences were observed among treatments for most meat quality traits (Table 4) and chemical composition variables (Table 5) (P > 0.05). The only exception was L*, which tended to be greater in animals fed MON than in those fed BEO + HyD (P = 0.10), whereas animals fed BEO showed intermediate values and did not differ from the other treatments.

**Table 4:** Effects of feed additives and their combinations on meat quality traits of feedlot F1 Angus-Nellore bulls.

Item^a^	Treatmentsb			SEM^c^	*P*-value^d^
	MON	BEO	BEO + HyD		
**pH**	5.39	5.33	5.50	0.07	0.26
**Shear force, kg, kg**	6.13	5.78	6.05	0.33	0.74
**Cooking loss, %**	27.56	27.87	28.87	0.86	0.55
L*	29.75^a^	28.49^ab^	27.24^b^	0.76	0.10
a*	18.90	17.78	17.85	0.80	0.55
b*	7.55	7.11	7.13	0.36	0.63
**Hue angle**	21.84	21.80	21.62	0.47	0.94
**Chroma**	20.35	19.15	19.22	0.86	0.55

^a^L = Lightness, a* = Redness, b* = Yellowness.

^b^MON = sodium monensin (26 mg/kg DM); BEO = blend of essential oils + exogenous α-amylase (90 and 560 mg/kg DM, respectively); BOE + HyD = blend of essential oils + exogenous α-amylase + 25- hydroxycholecalciferol (90, 560 mg/kg mg/kg DM and 1 mg/animal/day, respectively). Sodium monensin (Rumensin) was from Elanco Animal Health, Indianapolis, IN. The blend of essential oils (CRINA Ruminants), and the exogenous enzymes (α-amylase [RONOZYME RumiStar] and 25- hydroxycholecalciferol (Rovimix Hy-D 1.25%) were provided by DSM Nutritional Products, Basel, Switzerland.

^c^SEM: standard error of the mean.

^d^*P* ≤ 0.05 values were considered significant effects and trends were considered at 0.05 *< P ≤* 0.10. a-c: Means with different letters in the same row differ (*P <* 0.05).

**Table 5:** Effects of feed additives and their combinations on centesimal composition of the meat of feedlot F1 Angus-Nellore bulls.

Item, %	Treatments^a^			SEM^b^	*P*-value^c^
	MON	BEO	BEO + HyD		
**Moisture**	74.37	73.91	74.44	0.21	0.16
**Protein**	22.95	22.92	23.06	0.09	0.55
**Ether extract**	2.45	2.80	2.37	0.19	0.25
**Ashes**	1.11	1.09	1.06	0.02	0.18

^a^MON = sodium monensin (26 mg/kg DM); BEO = blend of essential oils + exogenous α-amylase (90 and 560 mg/kg DM, respectively); BOE + HyD = blend of essential oils + exogenous α-amylase + 25- hydroxycholecalciferol (90, 560 mg/kg mg/kg DM and 1 mg/animal/day, respectively). Sodium monensin (Rumensin) was from Elanco Animal Health, Indianapolis, IN. The blend of essential oils (CRINA Ruminants), and the exogenous enzymes (α-amylase [RONOZYME RumiStar] and 25- hydroxycholecalciferol (Rovimix Hy-D 1.25%) were provided by DSM Nutritional Products, Basel, Switzerland.

^b^SEM: standard error of the mean.

^c^*P* ≤ 0.05 values were considered significant effects and trends were considered at 0.05 *< P ≤* 0.10. a-c: Means with different letters in the same row differ (*P <* 0.05).

### Apparent total tract starch digestibility

Compared with MON, fecal starch concentration was reduced by 39.58% in BEO and 57.51% in BEO + HyD (P = 0.004). Similarly, total tract starch digestibility increased by 2.41% in BEO and 3.48% in BEO + HyD (P = 0.002). Fecal nitrogen was not affected by treatments (P = 0.11) (Table 6).

**Table 6:** Effects of feed additives and their combinations on fecal starch and apparent total tract digestibility of starch of feedlot F1 Angus-Nellore bulls.

Item, %	Treatments^a^			SEM^b^	*P-*Value^c^
	MON	BEO	BEO + HyD		
**Fecal starch**	11.39^a^	7.19^b^	4.84^b^	1.18	0.004
**Fecal nitrogen**	2.35	2.16	2.18	0.06	0.11
**Total tract starch digestion**	94.08^b^	96.35^a^	97.35^a^	0.56	0.002

^a^MON = sodium monensin (26 mg/kg DM); BEO = blend of essential oils + exogenous α-amylase (90 and 560 mg/kg DM, respectively); BOE + HyD = blend of essential oils + exogenous α-amylase + 25- hydroxycholecalciferol (90, 560 mg/kg mg/kg DM and 1 mg/animal/day, respectively). Sodium monensin (Rumensin) was from Elanco Animal Health, Indianapolis, IN. The blend of essential oils (CRINA Ruminants), and the exogenous enzymes (α-amylase [RONOZYME RumiStar] and 25- hydroxycholecalciferol (Rovimix Hy-D 1.25%) were provided by DSM Nutritional Products, Basel, Switzerland.

^b^SEM: standard error of the mean.

^c^*P* ≤ 0.05 values were considered significant effects and trends were considered at 0.05 *< P ≤* 0.10. a-c: Means with different letters in the same row differ (*P <* 0.05).

### Rumenitis score, rumen and cecum morphometrics

No treatment effects were observed for rumenitis score, number of papillae, or papillae area (P > 0.05; Table 7). However, animals fed BEO and MON showed greater ASA (P = 0.007) and papillae area expressed as a percentage of ASA (P = 0.01) compared with those fed BEO + HyD. No liver abscesses were observed in bulls slaughtered at the end of the experimental period. Additionally, no treatment effects were observed for cecal morphometric parameters (P > 0.05).

**Table 7:** Effects of feed additives and their combinations on rumen morphometrics of feedlot F1 Angus-Nellore bulls.

Item^a^	Treatments^b^			SEM^c^	*P*-value^d^
	MON	BEO	BEO + HyD		
**Rumen measurements**					
**Rumenitis score**	0.86	0.75	1.00	0.23	0.74
**Macroscopic variables**					
**No. of papillae (n)**	67.22	73.95	68.33	7.17	0.78
**Mean papillae area, cm** ^**2**^	0.51	0.50	0.43	0.04	0.46
**ASA, (cm**^**2**^**/cm**^**2**^ **of rumen wall)**	33.87^a^	36.12^a^	28.47^b^	1.58	0.007
**Papillae area (% of ASA)**	97.40^a^	97.60^a^	96.90^b^	0.16	0.01
**Papillae height, cm**	2.65	2.75	2.14	0.22	0.15
**Papillae width, mm**	0.70	0.72	0.95	0.08	0.11
**Papillae surface area, mm** ^**2**^	1.12^b^	1.49^a^	1.05^b^	0.12	0.04
**Keratinised layer thickness, μm**	5.39	6.41	5.76	0.53	0.38
**Cecum measurements**					
**Crypt depth, μm**	38.52	46.60	37.41	3.48	0.16
**Enterocytes, n**	18.50	19.76	15.94	1.62	0.30
**Goblet cells, n**	1.43	1.69	1.24	0.20	0.31
**Crypt/Enterocytes**	2.10	2.38	2.34	0.10	0.14
**Crypt/Goblet, n**	28.62	30.89	30.34	3.04	0.85

^a^ASA = absorptive surface area.

^b^MON = sodium monensin (26 mg/kg DM); BEO = blend of essential oils + exogenous α-amylase (90 and 560 mg/kg DM, respectively); BOE + HyD = blend of essential oils + exogenous α-amylase + 25- hydroxycholecalciferol (90, 560 mg/kg mg/kg DM and 1 mg/animal/day, respectively). Sodium monensin (Rumensin) was from Elanco Animal Health, Indianapolis, IN. The blend of essential oils (CRINA Ruminants), and the exogenous enzymes (α-amylase [RONOZYME RumiStar] and 25- hydroxycholecalciferol (Rovimix Hy-D 1.25%) were provided by DSM Nutritional Products, Basel, Switzerland.

^c^SEM: standard error of the mean.

^d^*P* ≤ 0.05 values were considered significant effects and trends were considered at 0.05 *< P ≤* 0.10. a-c: Means with different letters in the same row differ (*P <* 0.05).

### Ruminal pH

During the adaptation period (i.e., the first 14 days), animals fed BEO and BEO + HyD had greater mean ruminal pH (P = 0.001) and minimum ruminal pH (P < 0.0001) than those fed MON (Table 8). There was also a tendency for maximum ruminal pH (P = 0.09), with animals fed BEO showing greater values than those fed MON, whereas the BEO + HyD treatment was intermediate and did not differ from the other treatments.

**Table 8:** Effects of feed additives and their combinations on ruminal pH of feedlot F1 Angus-Nellore bulls during adaptation period.

Item^a^	Treatments^b^			SEM^c^	*P*-value^d^
	MON	BEO	BEO + HyD		
**Ruminal pH**					
**Minimum**	5.57^b^	5.89^a^	5.87^a^	0.05	<.0001
**Maximum**	6.68^b^	6.82 ^a^	6.76^ab^	0.06	0.09
**Mean**	6.16^b^	6.40^a^	6.36^a^	0.05	0.001
**Area under the curve, pH x min**					
**< 5,8**	41.64	33.06	29.08	7.92	0.55
**< 6,0**	109.91	75.03	79.75	17.47	0.28
**< 6,2**	235.64^a^	139.67^b^	169.71^ab^	28.88	0.05
**Duration pH, min/day**					
**< 5,8**	235.71	159.64	166.43	34.43	0.26
**< 6,0**	452.62^a^	256.07^b^	340.54^ab^	51.75	0.04
**< 6,2**	788.33^a^	400.89^b^	552.50^b^	63.42	0.0002
**Mean temperature, ˚C**	39.53^a^	39.42^b^	39.48^b^	0.03	0.05

^a^Variables AUC = area under the curve (dimensionless) and DUR = duration (min/day), under the given pH threshold.

^b^MON = sodium monensin (26 mg/kg DM); BEO = blend of essential oils + exogenous α-amylase (90 and 560 mg/kg DM, respectively); BOE + HyD = blend of essential oils + exogenous α-amylase + 25- hydroxycholecalciferol (90, 560 mg/kg mg/kg DM and 1 mg/animal/day, respectively). Sodium monensin (Rumensin) was from Elanco Animal Health, Indianapolis, IN. The blend of essential oils (CRINA Ruminants), and the exogenous enzymes (α-amylase [RONOZYME RumiStar] and 25- hydroxycholecalciferol (Rovimix Hy-D 1.25%) were provided by DSM Nutritional Products, Basel, Switzerland.

^c^SEM: standard error of the mean.

^d^*P* ≤ 0.05 values were considered significant effects and trends were considered at 0.05 *< P ≤* 0.10. a-c: Means with different letters in the same row differ (*P <* 0.05).

Animals fed MON tended to present a greater area under the curve below pH 6.2 (P = 0.05) compared with those fed BEO, whereas the BEO + HyD treatment was intermediate and did not differ from the other treatments. However, treatments did not affect the area under the curve below pH 5.8 (P = 0.55) or 6.0 (P = 0.28). Furthermore, animals fed BEO spent less time below pH 6.0 than those fed MON (P = 0.04), whereas the BEO + HyD treatment was intermediate and did not differ from the other treatments. The duration of ruminal pH below 6.2 was shorter in animals fed BEO and BEO + HyD compared with those fed MON (P = 0.0002). In contrast, treatments did not affect the duration of ruminal pH below 5.8 (P = 0.26).

Regarding ruminal temperature, animals fed MON had greater ruminal temperature than those fed BEO and BEO + HyD (P = 0.05).

Over the entire feeding period (Table 9), animals fed BEO had greater mean, minimum, and maximum ruminal pH values than those fed MON, whereas the BEO + HyD treatment showed intermediate values and differed from both MON and BEO (P < 0.0001).

**Table 9:** Effects of feed additives and their combinations on ruminal pH of feedlot F1 Angus-Nellore bulls during the total feeding period.

Item	Treatments^1^			SEM	*P*-value
	MON	BEO	BEO + HyD		
**pH ruminal**					
**Minimum**	5.60^c^	5.92^a^	5.85^b^	0.02	<.0001
**Maximum**	6.78^c^	7.08^a^	6.91^b^	0.02	<.0001
**Mean**	6.22^c^	6.51^a^	6.42^b^	0.02	<.0001
**Area under the curve, pH x min**					
**< 5,8**	26.79	22.99	23.39	2.72	0.59
**< 6,0**	81.52^a^	57.76^b^	60.96^b^	5.27	0.005
**< 6,2**	190.24^a^	118.64^b^	133.22^b^	8.82	<.0001
**pH duration under, min/d**					
**< 5,8**	161.10^a^	121.13^b^	122.56^b^	11.20	0.02
**< 6,0**	390.79^a^	228.51^b^	260.05^b^	16.96	<.0001
**< 6,2**	687.01^a^	379.69^c^	455.28^b^	22.21	<.0001
**Mean temperature, ˚C**	39.60^a^	39.51^b^	39.53^b^	0,.01	<.0001

^a^Variables AUC = area under the curve (dimensionless) and DUR = duration (min/day), under the given pH threshold.

^b^MON = sodium monensin (26 mg/kg DM); BEO = blend of essential oils + exogenous α-amylase (90 and 560 mg/kg DM, respectively); BOE + HyD = blend of essential oils + exogenous α-amylase + 25- hydroxycholecalciferol (90, 560 mg/kg mg/kg DM and 1 mg/animal/day, respectively). Sodium monensin (Rumensin) was from Elanco Animal Health, Indianapolis, IN. The blend of essential oils (CRINA Ruminants), and the exogenous enzymes (α-amylase [RONOZYME RumiStar] and 25- hydroxycholecalciferol (Rovimix Hy-D 1.25%) were provided by DSM Nutritional Products, Basel, Switzerland.

^c^SEM: standard error of the mean.

^d^*P* ≤ 0.05 values were considered significant effects and trends were considered at 0.05 *< P ≤* 0.10. a-c: Means with different letters in the same row differ (*P <* 0.05).

**Fig 2:**
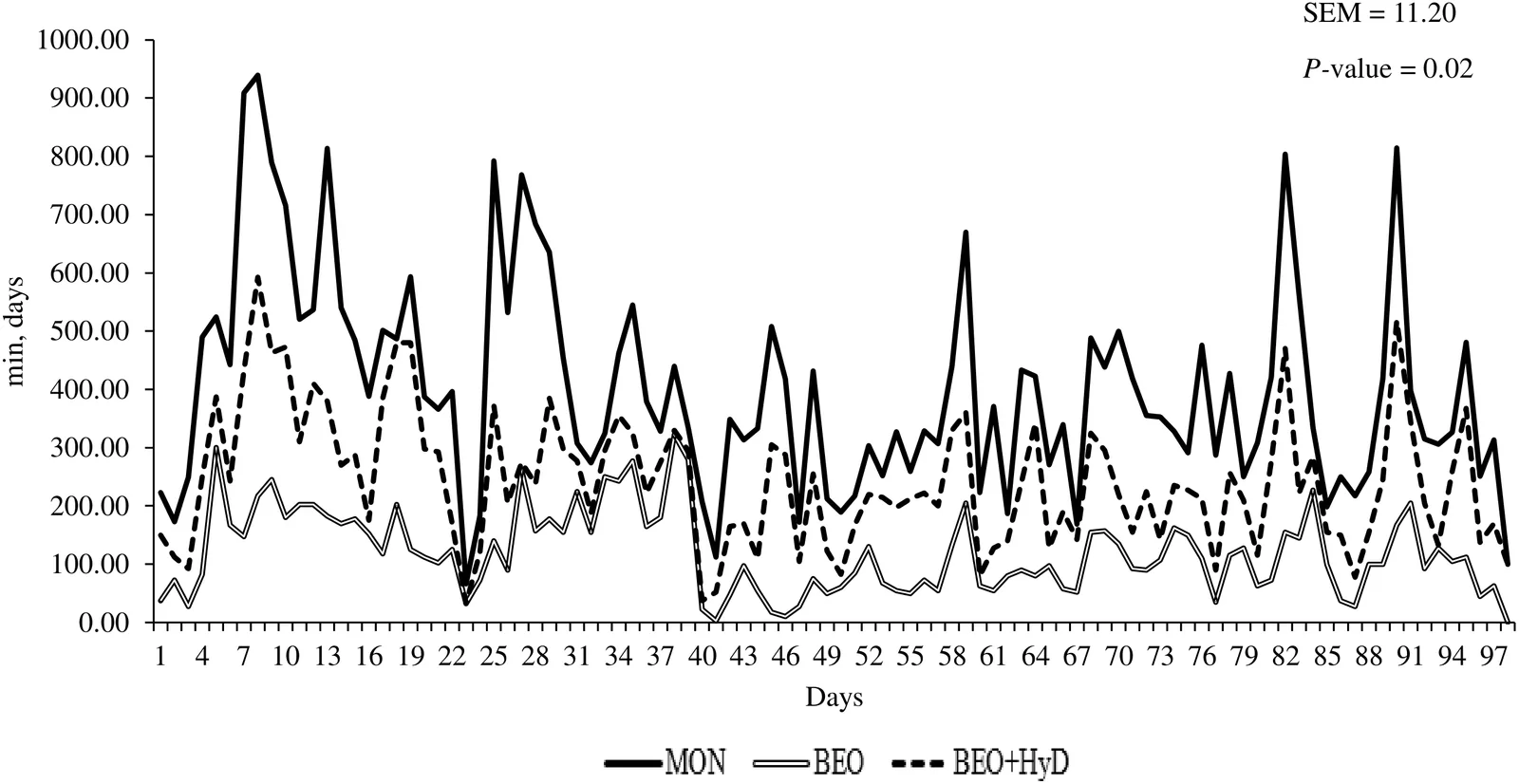
Effects of feed additives and their combinations on the daily duration of ruminal pH below 5.8 of feedlot F1 Angus-Nellore bulls during the total feeding period. Days 1–98 correspond to observations made during the total feeding period. MON = sodium monensin (26 mg/kg DM); BEO = blend of essential oils + exogenous α-amylase (90 and 560 mg/kg DM, respectively); BEO+HyD = blend of essential oils + exogenous α-amylase + 25- hydroxycholecalciferol (90, 560 mg/kg mg/kg DM and 1 mg/animal/day, respectively). Sodium monensin (Rumensin) was from Elanco Animal Health, Indianapolis, IN. The blend of essential oils (CRINA Ruminants), and the exogenous enzymes (α-amylase [RONOZYME RumiStar] and 25- hydroxycholecalciferol (Rovimix Hy-D 1.25%) were provided by DSM Nutritional Products, Basel, Switzerland. SEM: standard error of the mean. *P* ≤ 0.05 values were considered significant effects and trends were considered at 0.05 < P ≤ 0.10.

Furthermore, feeding BEO and BEO + HyD reduced the area under the curve below pH 6.0 (P = 0.005) and 6.2 (P < 0.0001), and decreased the duration of ruminal pH below 5.8 (P = 0.02; Fig 2), 6.0 (P < 0.0001), and 6.2 (P < 0.0001) compared with MON. Animals fed BEO also spent less time below pH 6.2 than those fed BEO + HyD and MON.

Regarding ruminal temperature, feeding MON increased ruminal temperature compared with BEO and BEO + HyD treatments (P < 0.0001). However, treatments did not affect the area under the curve below pH 5.8 (P = 0.59).

### Serum and muscle calcium, phosphorus and 25-(OH)D_3_ concentrations

No significant differences were observed among treatments for serum or muscle P concentrations or for muscle Ca concentrations (P > 0.05) (Table 10). However, animals fed BEO had greater serum tCa concentrations (P = 0.04) and tended to have greater iCa concentrations (P = 0.08) compared with those fed BEO + HyD, whereas animals fed MON showed intermediate values and did not differ from the other treatments. Serum concentrations of 25-(OH)D₃ were greater (P = 0.002) in animals fed BEO + HyD than in those fed MON and BEO.

**Table 10:** Effects of feed additives and their combinations serum and muscle concentrations of calcium, phosphorus and 25-(OH)D_3_ of feedlot F1 Angus-Nellore bulls.

Item	Treatments^a^			SEM^b^	*P*-value^c^
	MON	BEO	BEO + HyD		
**Serum**					
**Calcium, μg/mL**	74.19^ab^	77.82^a^	67.28^b^	2.17	0.04
**Phosphorus, μg/mL**	69.77	77.00	70.75	4.59	0.38
**Ionized Calcium, mg/dL**	2.43^ab^	4.38^a^	2.28^b^	0.68	0.08
**25-(OH)D**_**3**_ **, ng/mL**	16.28^b^	32.50^b^	67.40^a^	7.11	0.002
**Muscle**					
**Calcium, mg/100 g**	8.29	4.15	4.70	1.73	0.20
**Phosphorus, mg/100 g**	7.63	4.49	5.28	1.57	0.34

^a^MON = sodium monensin (26 mg/kg DM); BEO = blend of essential oils + exogenous α-amylase (90 and 560 mg/kg DM, respectively); BOE + HyD = blend of essential oils + exogenous α-amylase + 25- hydroxycholecalciferol (90, 560 mg/kg mg/kg DM and 1 mg/animal/day, respectively). Sodium monensin (Rumensin) was from Elanco Animal Health, Indianapolis, IN. The blend of essential oils (CRINA Ruminants), and the exogenous enzymes (α-amylase [RONOZYME RumiStar] and 25- hydroxycholecalciferol (Rovimix Hy-D 1.25%) were provided by DSM Nutritional Products, Basel, Switzerland.

^b^SEM: standard error of the mean.

^c^*P* ≤ 0.05 values were considered significant effects and trends were considered at 0.05 *< P ≤* 0.10. a-c: Means with different letters in the same row differ (*P <* 0.05).

### Gene expression

**Fig 3:**
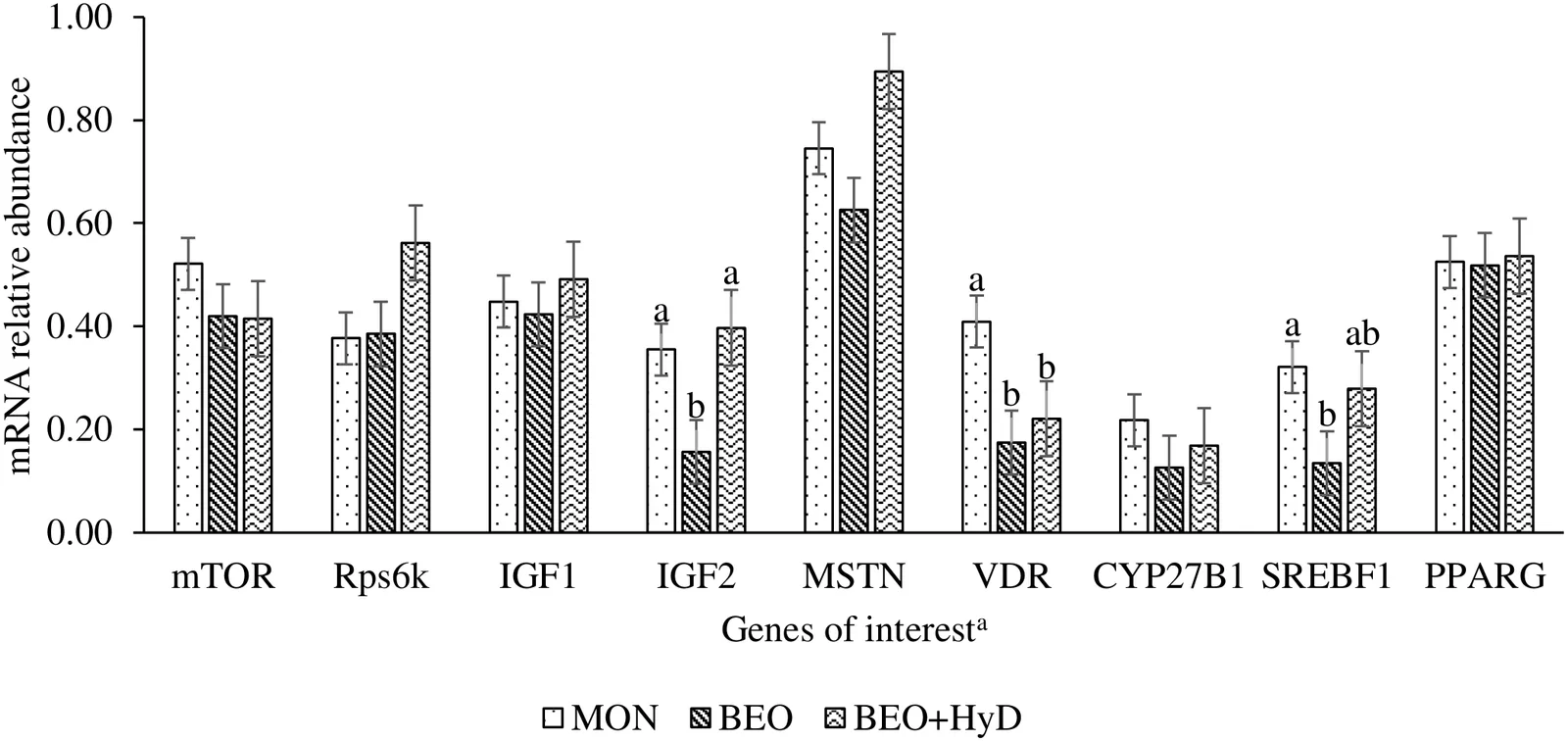
Effects of feed additives and their combinations gene expression in muscle of feedlot F1 Angus-Nellore bulls. MON (green bar) = sodium monensin (26 mg/kg DM); BEO (red bar) = blend of essential oils + exogenous α-amylase (90 and 560 mg/kg DM, respectively); BOE + HyD (blue bar) = blend of essential oils + exogenous α-amylase + 25-hydroxycholecalciferol (90, 560 mg/kg mg/kg DM and 1 mg/animal/day, respectively). Sodium monensin (Rumensin) was from Elanco Animal Health, Indianapolis, IN. The blend of essential oils (CRINA Ruminants), and the exogenous enzymes (α-amylase [RONOZYME RumiStar] and 25-hydroxycholecalciferol (Rovimix Hy-D 1.25%) were provided by DSM Nutritional Products, Basel, Switzerland. ^a^Genes include: mTOR (mechanistic target of rapamycin); Rps6k (ribosomal protein S6 kinase A1); IGF1 (insulin-like growth factor 1); IGF2 (insulin-like growth factor 2); MSTN (myostatin); VDR (vitamin D_3_ receptor); CYP27B1 (cytochrome P450 family 27 subfamily B member 1); SREBF1 (sterol regulatory element binding transcription factor 1); PPARG (peroxisome proliferator-activated receptor gamma). P ≤ 0.05 values were considered significant effects and trends were considered at 0.05 < P ≤ 0.10. a-b: Means with different letters within genes differ (P < 0.05). Treatment effects were significant for IGF2 (*P* = 0.0003), VDR (*P* = 0.01), and SREBF1 (*P* = 0.01), whereas no differences were observed for thengenes mTOR (*P* = 0.12), Rps6k (P = 0.27), IGF1 (*P* = 0.56), MSTN (*P* = 0.12), CYP27B1 (*P* = 0.21) and PPARG (*P* = 0.97).

**Fig 4:**
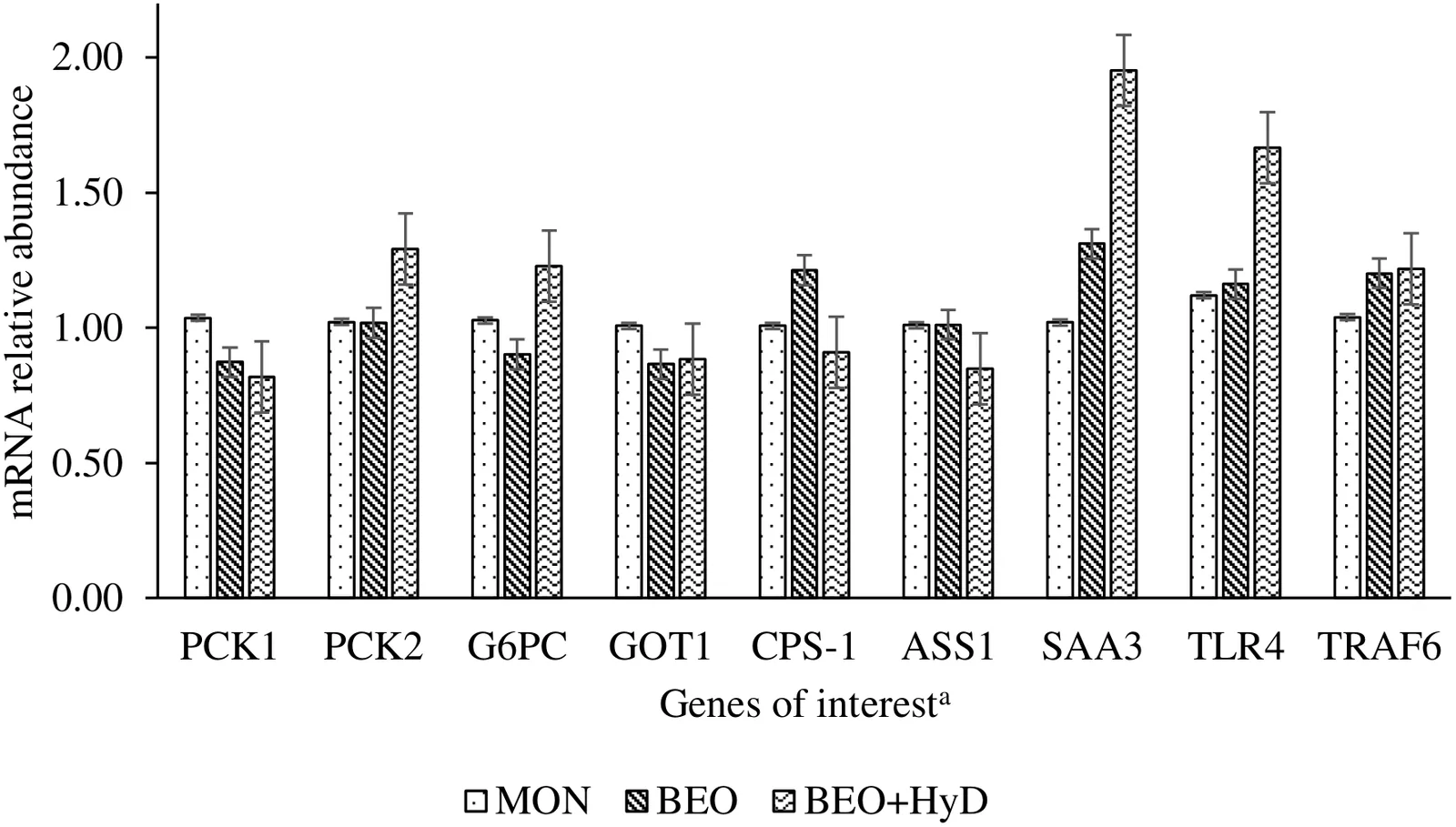
Effects of feed additives and their combinations gene expression in liver of feedlot F1 Angus-Nellore bulls. MON (green bar) = sodium monensin (26 mg/kg DM); BEO (red bar) = blend of essential oils + exogenous α-amylase (90 and 560 mg/kg DM, respectively); BOE + HyD (blue bar) = blend of essential oils + exogenous α-amylase + 25-hydroxycholecalciferol (90, 560 mg/kg mg/kg DM and 1 mg/animal/day, respectively). Sodium monensin (Rumensin) was from Elanco Animal Health, Indianapolis, IN. The blend of essential oils (CRINA Ruminants), and the exogenous enzymes (α-amylase [RONOZYME RumiStar] and 25-hydroxycholecalciferol (Rovimix Hy-D 1.25%) were provided by DSM Nutritional Products, Basel, Switzerland. ^a^Genes include: PCK1 (phosphoenolpyruvate carboxykinase 1); PCK2 (phosphoenolpyruvate carboxykinase 2); G6PC (glucose-6-phosphatase catalytic subunit); CPS-1 (carbamoyl-phosphate synthase 1); GOT1 (glutamic-oxaloacetic transaminase 1); ASS1 (argininosuccinate synthase 1); SAA3 (serum amyloid A3); TLR4 toll-like receptor 4 and TRAF6 (TNF receptor-associated factor). P ≤ 0.05 values were considered significant effects and trends were considered at 0.05 < P ≤ 0.10. a-b: Means with different letters within genes differ (P < 0.05). No treatment effects were observed for the genes PCK1 (P = 0.38), PCK2 (P = 0.34), G6PC (P = 0.36), GOT1 (P = 0.40), CPS-1 (P = 0.11) ASS1 (P = 0.46), SAA3 (P = 0.18), TLR4 (P = 0.71) and TRAF6 (P = 0.59).

Gene expression data for the LT muscle and liver are presented in Figs 3 and 4, respectively. In muscle tissue, differences among treatments were observed only for IGF2, VDR, and SREBF1. Animals fed MON and BEO + HyD showed greater IGF2 expression (P = 0.0003) than those fed BEO. In addition, MON increased the expression of VDR (P = 0.01) and SREBF1 (P = 0.01), whereas the BEO + HyD treatment showed intermediate values and did not differ from the other treatments for SREBF1 expression.

In liver tissue, no differences in gene expression were observed among treatments for any of the evaluated genes (P > 0.05; Fig 4).

## Discussion

This study evaluated the effects of replacing sodium monensin with alternative feed additive strategies based on essential oils, α-amylase, and 25-hydroxycholecalciferol on performance, ruminal environment, and digestive tract morphology in feedlot cattle fed high-concentrate diets. Considering that improvements in animal performance are closely associated with ruminal stability and nutrient utilization efficiency, the present results are discussed in terms of how these additives may modulate ruminal fermentation and digestive processes to enhance growth performance compared with monensin.

Given the central role of ruminal pH stability in maintaining fermentative efficiency and digestive health under high-concentrate feeding conditions, the present findings suggest that BEO-based strategies promoted a more stable ruminal environment than MON.

During the adaptation period (days 1–28), cattle fed BEO and BEO + HyD exhibited greater DMI than those fed MON, although no differences in performance were observed during this phase. Similar responses have been reported in feedlot cattle fed BEO during the early adaptation period, with greater DMI, which in some cases was associated with increased ADG and final BW, without changes in G:F [21,29]. The greater DMI observed in animals fed BEO and BEO + HyD during the adaptation phase may be considered a favorable response, as newly received feedlot cattle are typically subjected to stress and metabolic challenges that suppress feed intake. Therefore, the ability of these additives to promote earlier recovery of intake may have contributed to improved nutrient supply and subsequent growth performance during the finishing period.

Consistent with this initial response, animals fed BEO and BEO + HyD maintained greater DMI throughout the experimental period than those fed MON, resulting in greater carcass production despite similar feed efficiency among treatments. Similar responses have been reported for BEO-based strategies, which generally increase DMI and carcass weight without consistent effects on feed efficiency [21,23,29]. In some studies, greater intake was associated with increased ADG and hot carcass weight (HCW) [21,22], whereas others reported no differences in performance or carcass traits compared with MON [28,69], indicating that responses may vary according to experimental conditions.

In the present study, the greater performance observed in animals fed BEO and BEO + HyD appears to be associated not only with increased nutrient intake, but also with improved starch digestibility and utilization. These treatments showed lower fecal starch concentrations and greater total tract starch digestibility compared with MON, indicating more efficient utilization of dietary starch and, consequently, greater availability of metabolizable energy to support growth and carcass deposition. Reductions in fecal starch concentration and improvements in starch utilization have also been reported in cattle fed BEO-based additives [21,22], although responses are not always consistent across studies [23]. In addition, previous studies have demonstrated that BEO supplementation may increase ruminal starch digestibility compared with ionophore-based strategies [70]. In this context, the inclusion of α-amylase likely played an important role in these responses, as previous studies have shown that performance improvements associated with this enzyme are frequently related to increased nutrient availability and enhanced starch digestion [17].

Supplementation with α-amylase may have contributed to these responses, as exogenous amylolytic enzymes can enhance starch hydrolysis and increase the availability of fermentable substrates for ruminal microorganisms. The release of intermediate products, such as maltodextrins, may promote cross-feeding interactions between amylolytic and non-amylolytic bacteria, thereby improving overall nutrient digestion [16,71,72]. In addition, enhanced starch hydrolysis may favor fermentation pathways associated with greater propionate production and energetic efficiency, which may have contributed to the greater carcass production observed in animals fed BEO and BEO + HyD.

Importantly, the greater starch digestibility observed in these treatments was not accompanied by reductions in ruminal pH or signs of impaired digestive health. These findings indicate that the combination of essential oils and α-amylase improved starch utilization without compromising ruminal fermentative stability. Although the inclusion of 25-(OH)D₃ did not result in additional improvements in performance, its potential metabolic and molecular effects are discussed in the following sections.

According to Barker et al. [73], increased substrate supply to the rumen enhances microbial fermentation and short-chain fatty acid (SCFA) production, making efficient absorption essential to prevent excessive accumulation and the onset of acidosis. In the present study, absorptive surface area (ASA) and papillary area (% of ASA) did not differ between the MON and BEO treatments; however, papillary surface area was greater in animals fed BEO, suggesting localized structural adaptations of the ruminal epithelium. Silva et al. [29] reported greater ASA, papillary area (% of ASA), and papillae height in cattle fed BEO compared with MON, which is not entirely consistent with the findings of the present study, indicating that the response may depend on experimental conditions.

Essential oils have been shown to modulate ruminal fermentation patterns, particularly by increasing propionate production [74], a key SCFA involved in stimulating ruminal papillae development [75]. The greater dry matter intake (DMI) observed in the BEO treatment may have increased ruminal fermentation and SCFA production; however, the greater papillary development likely enhanced SCFA absorption, contributing to a more stable ruminal environment. Importantly, rumenitis scores were similar among treatments, indicating that none of the additive strategies adversely affected ruminal health. Collectively, these responses suggest improved ruminal function, which may have contributed to the greater growth performance observed in animals fed BEO.

Although animals fed BEO + HyD showed lower ASA compared with the other treatments, this reduction did not impair animal performance. While this response may suggest reduced absorptive capacity, the similar rumenitis scores and papillae keratinised layer thickness among treatments do not support an interpretation based on greater ruminal damage. Previous studies have indicated that rumenitis may reduce ASA and impair SCFA absorption [76], but such effects were not clearly evident under the conditions of the present study. Animals fed MON exhibited ASA values similar to those of the BEO treatment; however, the lower DMI and consequently lower NDF intake observed in this group may have reduced rumination and saliva secretion, potentially limiting buffering capacity and contributing to lower ruminal pH values compared with the other treatments.

The greater ruminal stability observed in animals fed BEO and BEO + HyD was further supported by the higher ruminal pH, reduced time below critical thresholds, and lower severity of subacute ruminal acidosis (SARA). Ruminal pH values below 5.8 are associated with impaired fiber digestion and increased risk of metabolic disorders [77–79], and both the duration and magnitude of pH depression are important indicators of SARA severity [80,81]. In the present study, animals fed BEO and BEO + HyD maintained higher ruminal pH values and spent less time below these thresholds, indicating a more stable fermentation pattern. This effect may be related to the ability of essential oils to modulate ruminal microbiota and reduce acid accumulation under high-starch diets [13]. Previous studies evaluating BEO-based strategies have reported inconsistent effects on ruminal pH. While some authors observed no differences compared with MON [21,23,70], others reported reduced time below low pH thresholds or greater mean ruminal pH in animals supplemented with essential oils [69,82]. This variability suggests that the response may depend on diet composition, intake level, and experimental conditions.

In the present study, BEO and BEO + HyD clearly improved ruminal pH stability compared with MON. Despite the greater DMI observed in these treatments, no alterations in cecal morphometry were detected, suggesting that the increased flow of fermentable substrates to the hindgut did not compromise cecal integrity. Previous studies have reported that BEO supplementation may modulate intestinal morphology and reduce inflammatory responses, even under high-starch feeding conditions [29,83], supporting the absence of detrimental effects observed in the present study.

Collectively, the combination of greater feed intake, improved starch digestibility, and enhanced ruminal pH stability likely contributed to a more efficient digestive process and to the greater performance observed in animals fed BEO and BEO + HyD. Furthermore, the maintenance of cecal integrity indicates that these improvements in ruminal function did not result in excessive post-ruminal fermentation, reinforcing that the increased nutrient utilization occurred without compromising digestive tract health.

During episodes of subacute ruminal acidosis (SARA), inflammatory responses may be increased due to elevated concentrations of free lipopolysaccharides (LPS) in the bloodstream, leading to greater hepatic expression of immune-related genes involved in inflammatory pathways, such as SAA3, TLR4 [84,85], and TRAF6 [86]. In the present study, however, no differences were observed in the expression of these genes among treatments, despite the differences in ruminal pH. This indicates that the magnitude of pH reduction was not sufficient to trigger systemic inflammatory responses or impair liver function.

In addition, although animals fed MON spent more time at ruminal pH values below 5.8, no changes were observed in the expression of lipogenic genes (SREBF1 and PPARG) or in carcass fat deposition. These findings suggest that the alterations in ruminal fermentation were not severe enough to affect lipid metabolism at the tissue level, as also reported in studies evaluating moderate diet-induced changes in ruminal fermentation [87,88]. Collectively, these results indicate that differences in ruminal pH among treatments did not result in measurable metabolic or inflammatory alterations.

Considering the greater dry matter intake and improved performance observed in animals fed BEO and BEO + HyD, it was hypothesized that hepatic expression of genes related to gluconeogenesis (PCK1, PCK2, and G6PC) would be increased, whereas genes associated with ureagenesis (CPS1, GOT1, and ASS1) would be reduced. However, this hypothesis was not confirmed. Despite evidence that increased propionate availability may stimulate the expression of gluconeogenic genes in ruminants [89] and reduce hepatic ureagenesis [90], no differences were observed among treatments. These findings suggest that the improved performance of animals fed BEO and BEO + HyD was not driven by transcriptional changes in hepatic energy metabolism, but rather by increased nutrient intake and improved digestive efficiency.

The greater carcass production observed in the BEO and BEO + HyD treatments during the finishing phase was attributed to muscle hypertrophy, as an increase in muscle fiber cross-sectional area was observed in the LT muscle. To the best of our knowledge, this is the first study to demonstrate an increase in mean muscle fiber area in cattle supplemented with this specific combination of BEO or BEO + HyD. Likewise, supplementation with 25-(OH)D₃ has been reported to enhance muscle development and the expression of genes associated with protein synthesis [24,91,92]; however, no additional effects were observed in the present study, despite numerical improvements in carcass traits. This lack of effect may be partially associated with methodological limitations, including the relatively small number of experimental units, which may have limited the detection of subtle treatment responses. In addition, the absence of initial ultrasound evaluation and reference slaughter limited a more robust assessment of carcass development throughout the experimental period. Therefore, future studies using a larger number of animals, initial ultrasound evaluation, and baseline carcass measurements are needed to more comprehensively elucidate the effects of 25-(OH)D₃ on muscle growth and performance in feedlot cattle.

Overall, the absence of consistent changes in gene expression suggests that the performance improvements observed in animals fed BEO and BEO + HyD were primarily driven by improvements in digestive and metabolic efficiency rather than by direct modulation of gene expression pathways.

In addition to its role in Ca and P homeostasis [93], vitamin D has been widely investigated as a strategy to improve meat tenderness in beef cattle. Although supplementation with 25-(OH)D₃ increased circulating 25-(OH)D₃ concentrations, no corresponding changes were observed in serum or muscle Ca and P concentrations or in meat tenderness. Similar responses have been reported in the literature, in which increases in circulating 25-(OH)D₃ concentrations do not consistently translate into greater Ca and P availability or improvements in meat tenderness [94–96]. Although some studies have reported increases in serum or muscle Ca concentrations following 25-(OH)D₃ supplementation [24,91], these responses have not been consistently associated with reductions in shear force. In particular, strategies involving higher doses administered shortly before slaughter (24–48 h) have been proposed to enhance Ca mobilization and potentially improve meat tenderness [97]. This variability suggests that the effectiveness of vitamin D supplementation may depend on factors such as dose, duration, and timing of administration relative to slaughter [98]. However, under the conditions of the present study, supplementation with 25-(OH)D₃ did not result in sufficient increases in Ca availability to influence muscle proteolysis or meat quality traits.

In the present study, the additives had minimal effects on meat quality traits, with the exception of L*, which tended to be greater in animals fed MON. A similar response was reported by Toseti et al. [22], who also observed greater L* values in cattle fed MON compared with BEO. According to Abularach et al. [99], L* values below 29.7 indicate darker meat, whereas values above 38.5 are associated with lighter meat; therefore, the L* values observed in the present study suggest relatively darker meat, which may be less attractive to consumers.

Overall, previous studies have reported inconsistent effects of enzyme- and essential oil-based additives on meat quality traits. While some studies observed no changes in carcass pH, fat deposition, or color parameters, others reported improvements in redness or reductions in shear force depending on the additive type and experimental conditions [10,16,100,101]. This variability suggests that the effects of these additives on meat quality are limited and highly dependent on diet composition and management factors.

In the present study, the absence of consistent changes across most meat quality traits indicates that the improvements observed in performance and carcass production were not accompanied by major alterations in meat quality.

## Conclusion

Overall, the combination of essential oils and α-amylase improved ruminal stability and starch utilization, resulting in greater dry matter intake, carcass production, and overall performance in feedlot cattle fed high-concentrate diets. Importantly, these improvements were achieved without detrimental effects on cecal morphology or meat quality, indicating that the enhanced nutrient utilization did not compromise digestive tract health. Supplementation with 25-(OH)D₃ showed limited additional effects on performance and carcass traits under the conditions of the present study. Collectively, these findings suggest that the combination of essential oils and α-amylase represents a viable alternative to monensin by improving ruminal function and nutrient utilization efficiency in finishing feedlot cattle.

## Supporting information

S1 Table The nucleotide sequence of the housekeeping PCR primers used to assay gene expression by real-time quantitative PCR. ^a^ACTB = actin beta; GAPDH = glyceraldehyde-3-phosphate dehydrogenase. ^b^Primer direction. F= forward; R= Reverse. ^c^Amplicon Size in base pairs (bp). ^d^NCBI, National Center for Biotechnology Information database (www.ncbi.nlm.nih.gov).
(DOCX)

S2 Table The nucleotide sequence of the PCR primers used to assay gene expression by real-time quantitative PCR in liver tissue. ^a^PCK1 (phosphoenolpyruvate carboxykinase 1); PCK2 (phosphoenolpyruvate carboxykinase 2); G6PC (glucose-6-phosphatase catalytic subunit); CPS-1 (carbamoyl-phosphate synthase 1); GOT1 (glutamic-oxaloacetic transaminase 1); ASS1 (argininosuccinate synthase 1); SAA3 (serum amyloid A3); TLR4 toll-like receptor 4 and TRAF6 (TNF receptor-associated factor). ^b^Primer direction. F= forward; R= Reverse. ^c^Amplicon Size in base pairs (bp). ^d^NCBI, National Center for Biotechnology Information database (www.ncbi.nlm.nih.gov).
(DOCX)

S3 Table The nucleotide sequence of the PCR primers used to assay gene expression by real-time quantitative PCR in muscle tissue. ^a^mTOR (mechanistic target of rapamycin); RPS6KA1 (ribosomal protein S6 kinase A1); IGF1 (insulin-like growth factor 1); IGF2 (insulin-like growth factor 2); MSTN (myostatin); VDR (vitamin D_3_ receptor); CYP27B1 (cytochrome P450 family 27 subfamily B member 1); SREBF1 (sterol regulatory element binding transcription factor 1); PPARG (peroxisome proliferator-activated receptor gamma). ^b^Primer direction. F= forward; R= Reverse. ^c^Amplicon Size in base pairs (bp). ^d^NCBI, National Center for Biotechnology Information database (www.ncbi.nlm.nih.gov).
(DOCX)

S4 Fig Representative images of muscle fiber cross-sectional area for each treatment group: (A) MON, (B) BEO, and (C) BEO + HyD. I**mages were obtained from the *Longissimus thoracis* muscle using light microscopy.** MON = sodium monensin (26 mg/kg DM); BEO = blend of essential oils + exogenous α-amylase (90 and 560 mg/kg DM, respectively); BEO + HyD = blend of essential oils + exogenous α-amylase + 25- hydroxycholecalciferol (90, 560 mg/kg mg/kg DM and 1 mg/animal/day, respectively). Sodium monensin (Rumensin) was from Elanco Animal Health, Indianapolis, IN. The blend of essential oils (CRINA Ruminants), and the exogenous enzymes (α-amylase [RONOZYME RumiStar] and 25- hydroxycholecalciferol (Rovimix Hy-D 1.25%) were provided by DSM Nutritional Products, Basel, Switzerland.
(TIF)
