# Supplementary material for: Effects of selected feed additives on rumen pH dynamics, physiological responses, and rumen and cecum morphometrics in feedlot beef cattle
Source: PLoS One. 2026 Aug 19;21(8):e0356262. doi: 10.1371/journal.pone.0356262 (PMC13489448; doi:10.1371/journal.pone.0356262)
Supplement: Supplementary file 1 [file pone.0356262.s001.docx]

**Supporting Information**

**S1 Table. The nucleotide sequence of the housekeeping PCR primers used to assay gene expression by real-time quantitative PCR.**

| Target Gene^a^ |  | Primers^b^ | bp^c^ | NCBI accession No.^d^ |
| --- | --- | --- | --- | --- |
| ACTB |  | F: ATATTGCTGCGCTCGTGGTC | 149 | NM_173979.3 |
|  |  | R: GTACGAGTCCTTCTGGCCCAT |  |  |
| GAPDH |  | F: AGCCGTAACTTCTGTGCTGT | 148 | NM_001034034.2 |
|  |  | R: GAAGGGGTCATTGATGGCGA |  |  |

^a^ACTB = actin beta; GAPDH = glyceraldehyde-3-phosphate dehydrogenase.

^b^Primer direction. F= forward; R= Reverse.

^c^Amplicon Size in base pairs (bp).

^d^NCBI, National Center for Biotechnology Information database (www.ncbi.nlm.nih.gov).
