# Supplementary material for: Effects of selected feed additives on rumen pH dynamics, physiological responses, and rumen and cecum morphometrics in feedlot beef cattle
Source: PLoS One. 2026 Aug 19;21(8):e0356262. doi: 10.1371/journal.pone.0356262 (PMC13489448; doi:10.1371/journal.pone.0356262)
Supplement: Supplementary file 2 [file pone.0356262.s002.docx]

**Supporting Information**

**S2 Table. The nucleotide sequence of the PCR primers used to assay gene expression by real-time quantitative PCR in liver tissue.**

| Target Gene^a^ |  | Primers^b^ | bp^c^ | NCBI accession No.^d^ |
| --- | --- | --- | --- | --- |
| PCK1 |  | F: TTCGTCAAGTGCCTCCACTC | 126 | NM_174737.2 |
|  |  | R: GGAGATGATTTCTCGGCGGT |  |  |
| PCK2 |  | F: TATGAGGCCTTCAACTGGCG | 150 | NM_001205594.1 |
|  |  | R: AAGGTAGCGCCCAAAGTTGT |  |  |
| G6PC |  | F: TTCGAGAAGCTGTGGGCATC | 109 | NM_001076124.2 |
|  |  | R: AAAACCCACCAGTATGGGCG |  |  |
| CPS-1 |  | F: TGGATGCTGTCGGCAAAGAT | 64 | NM_001192258.1 |
|  |  | R: GCATCCTCCACGTGTTCAGA |  |  |
| GOT1 |  | F: CGGAATGTTCAGCTTCACCG | 104 | NM_177502.2 |
|  |  | R: AGCCACACATGTTGATCCGA |  |  |
| ASS1 |  | F: CCGCAACGATCTGATGGAGT | 120 | KF933365.1 |
|  |  | R: TCCAGGATTCCAGCCTCGTA |  |  |
| SAA3 |  | F: GCCAGAGATGGGGGACATTC | 119 | NM_181016.3 |
|  |  | R: ACGGGCGTGGAAGTATTTGT |  |  |
| TLR4 |  | F: CCTGAGACATTTAGGCAGCCA | 109 | MT424003.1 |
|  |  | R: TAGGCCCTGAAATGTGTCGTC |  |  |
| TRAF6 |  | F: ACTCTGAGAACAAAGGGCCAA | 125 | NM_001034661.2 |
|  |  | R: CAACAGTCGCTCTCGGACT |  |  |

^a^PCK1 (phosphoenolpyruvate carboxykinase 1); PCK2 (phosphoenolpyruvate carboxykinase 2); G6PC (glucose-6-phosphatase catalytic subunit); CPS-1 (carbamoyl-phosphate synthase 1); GOT1 (glutamic-oxaloacetic transaminase 1); ASS1 (argininosuccinate synthase 1); SAA3 (serum amyloid A3); TLR4 toll-like receptor 4 and TRAF6 (TNF receptor-associated factor).

^b^Primer direction. F= forward; R= Reverse.

^c^Amplicon Size in base pairs (bp).

^d^NCBI, National Center for Biotechnology Information database (www.ncbi.nlm.nih.gov).
