# Supplementary material for: Effects of selected feed additives on rumen pH dynamics, physiological responses, and rumen and cecum morphometrics in feedlot beef cattle
Source: PLoS One. 2026 Aug 19;21(8):e0356262. doi: 10.1371/journal.pone.0356262 (PMC13489448; doi:10.1371/journal.pone.0356262)
Supplement: Supplementary file 3 [file pone.0356262.s003.docx]

**Supporting Information**

**S3 Table. The nucleotide sequence of the PCR primers used to assay gene expression by real-time quantitative PCR in muscle tissue.**

| Target Gene^a^ |  | Primers^b^ | bp^c^ | NCBI accession No.^d^ |
| --- | --- | --- | --- | --- |
| mTOR |  | F: ATGTGCGAACACAGCAACAC | 146 | XM_015466778.1 |
|  |  | R: CCTTTCACGTTCCTCTCCCC |  |  |
| RPS6KA1 |  | F: GACCCGGAGAATGGTCAAGC | 104 | NC_037329.1 |
|  |  | R: CCAGCCTTGACATGGTGTGT |  |  |
| IGF1 |  | F: GACATGCCCAAGGCTCAGAA | 112 | NM_001077828.1 |
|  |  | R: CCTCCTTCTGTTCCCCTCCT |  |  |
| IGF2 |  | F: CAGTGATCTGTCCGTGGGCT | 114 | NM_174087.3 |
|  |  | R: CCTCCTCGTCCTCAAGGTCA |  |  |
| MSTN |  | F: CCAGGACGGAAACGGTCATTA | 70 | NM_001001525.3 |
|  |  | R: TTGGGTTTTCCTTCCACTTGC |  |  |
| VDR |  | F: CCAGCTACCTGTCCTGGGAT | 83 | NM_001167932.2 |
|  |  | R: CACCACCATTTGGCTCCTCT |  |  |
| CYP27B1 |  | F: ATCTCTCCCCTTCGGCTTTG | 149 | NM_001192284.1 |
|  |  | R: CAGTCCGGGTCATTGGTCTG |  |  |
| SREPB1 |  | F: CCGACATAGAAGGTGCGTCA | 126 | GCF_002263795.3 |
|  |  | R: GCATGTCTTCGAACGTGCAG |  |  |
| PPARG |  | F: TTCCGTTCCCAAGAGCTGAC | 98 | NM_181024.2 |
|  |  | R: TGGGGATACAGGCTCCACTT |  |  |

^a^mTOR (mechanistic target of rapamycin); RPS6KA1 (ribosomal protein S6 kinase A1); IGF1 (insulin-like growth factor 1); IGF2 (insulin-like growth factor 2); MSTN (myostatin); VDR (vitamin D_3_ receptor); CYP27B1 (cytochrome P450 family 27 subfamily B member 1); SREBF1 (sterol regulatory element binding transcription factor 1); PPARG (peroxisome proliferator-activated receptor gamma).

^b^Primer direction. F= forward; R= Reverse.

^c^Amplicon Size in base pairs (bp).

^d^NCBI, National Center for Biotechnology Information database ([www.ncbi.nlm.nih.gov](http://www.ncbi.nlm.nih.gov)).
